# Metal-catalyzed copolymerizations of epoxides and carbon disulfide for high-refractive index low absorbance adhesives and plastics

**DOI:** 10.3389/fchem.2023.1287528

**Published:** 2023-11-02

**Authors:** Derek B. Schwarz, Anvay Patil, Saranshu Singla, Ali Dhinojwala, James M. Eagan

**Affiliations:** School of Polymer Science and Polymer Engineering, The University of Akron, Akron, OH, United States

**Keywords:** metal catalyzed polymerization, epoxide copolymers, optics, complex refractive index, plastics

## Abstract

High-refractive index plastics are useful materials due to their optical properties, ease of processing, and low-costs compared to their inorganic counterparts. Catalytic carbon disulfide (CS_2_) copolymerization with epoxides is one method for producing low-cost high refractive index polymers. The reaction is accompanied by an oxygen-sulfur exchange reaction which produces irregular microstructures in the repeating units. In this study, metal salen catalysts were investigated with different metal centers (Al, Cr, Co) and salen ligand electronics, sterics, backbones, and co-catalyst in the copolymerization of CS_2_ with propylene oxide (PO) and cyclohexene oxide (CHO). The results reveal the essential nature of Cr metal centers on reactivity and the backbone geometry on monomer selectivity. There were no significant impacts on the O-S exchange reaction when ligand design changed, however PO and CHO/CS_2_ copolymers yield different monothiocarbonate microstructures. Additionally, the effects of microstructure on optical and thermal properties were investigated using spectroscopic ellipsometry and calorimetry, respectively. The CHO system produced high *T*_g_ plastics (93°C) with high refractive indexes (*n* up to 1.64), modest absorbance (*κ* < 0.020), and Abbe numbers of 32.2 while PO yielded low *T*_g_ adhesives (*T*_g_ = 9°C) with high refractive indexes (*n* up to 1.73), low absorbance (*κ* < 0.005), and low Abbe numbers (*V*_D_ = 19.1).

## 1 Introduction

High-refractive index materials are essential components for imaging technologies and optics (Vollmer and Mollmann, 2017). Infrared (IR) imaging systems, for example, are commercially derived from inorganic composites such as metal fluorides, or metal chalcogenides (e.g., Ge, Si, Cd, Ga, Zn); however, advances in high refractive index polymers opens the possibility of flexible, processible, and cost-efficient alternatives (Kleine et al., 2020). In order to deploy these active components in devices, they must adhere to detectors or multilayers and also match refractive indices with other components. In addition to controlling refractive index (*n*), the extinction coefficient of absorption (*κ*) should be low across the optical window to lessen potential signal interference. Polymerization strategies for affording high refractive index polymers primarily focus on propagation of monomers such as bromo-styrene, carbazole, and/or sulfur atom-containing moieties (Liu and Ueda, 2009; Zhou et al., 2023). Epoxides are abundant, low-cost monomers that serve as feedstocks for polyol precursors to polyurethane adhesives and plastics. Copolymerization of epoxides with sulfur containing comonomers such as COS (Luo et al., 2015; Wu et al., 2016; Yue et al., 2016; Ren et al., 2017; Yang et al., 2017; Zhang et al., 2018a; Zhang et al., 2018b; Yue et al., 2018; Sun et al., 2020; Wang et al., 2022) and CS_2_ (Zhang et al., 2008; Darensbourg et al., 2009; Zhang et al., 2009; Darensbou et al., 2013; Yang et al., 2020; McGuire and Buchard, 2021; Yang et al., 2021; Zhang et al., 2021) is an emerging approach to producing high-refractive index plastics.

**FIGURE 1:**
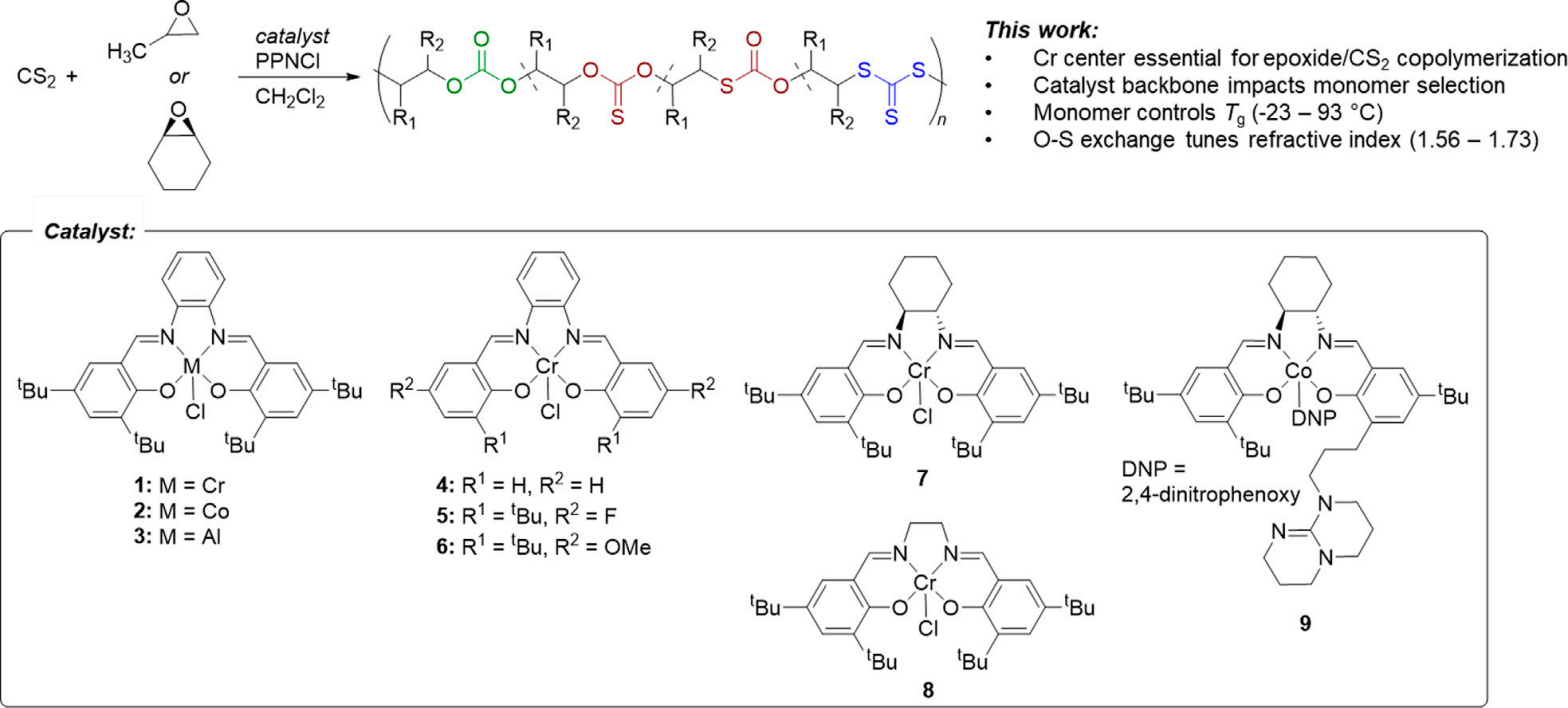
Comparative study of PO and CHO copolymerization with CS_2_ using variations on the salen ligand, metal center, and cocatalyst.

While episulfide/CS_2_ copolymerization produces well-defined trithiocarbonate repeat units (Nakano et al., 2007), epoxides introduce oxygen-sulfur exchange reactions that afford multiple thiocarbonate microstructures (Figure 1). Zhang reported this O-S exchange in double metal cyanide (DMC) catalyzed reactions between propylene oxide (PO) and CS_2_ (Zhang et al., 2008; Zhang et al., 2009), and Darensbourg observed the exchange in cyclohexene oxide (CHO) and CS_2_ using a chromium (III) salen catalyst (Darensbourg et al., 2009). Mechanistically, O-S exchange occurs either in the monomer or at the active chain-end (Zhang et al., 2008; Darensbourg et al., 2009; Yang et al., 2020). If catalyst systems can be devised that are capable of tuning the degree of O-S exchange, both the optical and thermo-mechanical properties of epoxide/CS_2_ copolymers could be tuned to access high *T*_g_ plastics or low *T*_g_ adhesives for optical devices. Since the catalyst is involved in both exchange and propagation pathways, we hypothesized that catalyst design may be a method for tuning the O-S exchange and therefore the optical properties of the produced polymers, while the monomer selection (i.e., CHO or PO) could afford access to high *T*_g_ plastics or low *T*_g_ adhesives.

Previous approaches to controlling O-S exchange include reaction temperature and [CS_2_]: [epoxide] which also bias the yield of cyclic and polymeric products (Darensbourg et al., 2009). Through control over O-S exchange chemistry, different thermal properties are obtained due to the crystallinity differences between polycarbonate and polythiocarbonate repeat units. Zhang and coworkers accessed a range of epoxide/CS_2_ thermal properties through the copolymerization of ethylene oxide with CS_2_ (Yang et al., 2021). In their system, metal-free Lewis acid/base pairs were used to produce semicrystalline blocky materials with varying degrees of O-S exchange and *T*_m_s as high as 245°C. The resulting semi-crystalline poly (ethylene trithiocarbonate) and poly (ethylene monothiocarbonate) linkages are segmented within low *T*_g_ poly (ethylene carbonate) (*T*_g_ = −35 to −18°C) and afford controlled degrees of crystallinity (0%–78% crystallinity). Darensbourg and coworkers reported the use of cyclopentene oxide/CS_2_ copolymerization using a Cr salen catalyst, which yielded O-S exchange microstructures and episulfide homopolymer linkages (i.e., polythioether) with low *T*_g_s (−17°C–32°C) (Darensbou et al., 2013). Werner and coworkers discovered propylene oxide, butylene oxide, and cyclohexene oxide copolymerize with CS_2_ in the presence of LiOtBu initiators to produce regioirregular polythiocarbonates with high molecular weights (up to 109 kg/mol) (Diebler et al., 2016). Through NMR spectroscopic analysis of the polythiocarbonates, the regioirregular head-to-head arrangements could be detected in thiocarbonate and tail-to-tail arrangements in the trithiocarbonate linkages.

As part of our interests in catalyst design, epoxide copolymerization, and polymer properties we herein investigate the effects of metal salen active site, sterics, electronics, and geometries on the O-S exchange reaction, polymerization activity, and monomer selectivity. The catalyst design enables the synthesis of either high *T*_g_ CHO/CS_2_ copolymers (93°C) or low *T*_g_ (−23°C) PO/CS_2_ copolymers that exhibit low absorbance and high refractive indices across the visible spectrum.

## 2 Materials and methods

### 2.1 Materials

CHO and PO were purified over CaH_2_ for 24 h, followed by vacuum transfer and degassing by three freeze-pump-thaw cycles. CH_2_Cl_2_ was purified through a solvent purification column equipped with molecular sieves. All catalysts were prepared using literature procedures (Hosseini Nejad et al., 2012) which followed salicylaldehyde condensation onto diamine backbones using formic acid in methanol, and subsequent metalation with Et_2_AlCl, CoCl_2_, or CrCl_2_ accordingly (Hosseini Nejad et al., 2012). Tethered binuclear catalyst **9** was synthesized according to the procedure of Lu and coworkers (Ren et al., 2009). Additional material details and the experimental spectra are available in the Supplementary Material.

### 2.2 Polymerization procedures

PO/CS_2_ copolymerization: a 25 mL Schlenk flask was dried in the oven overnight, brought into glovebox, and catalyst (0.01 mmol, 1.0 equiv.) and bis (triphenylphosphine) iminium chloride (0.01 mmol, 1.0 equiv.) were added. The solids were dissolved in 2 mL of CH_2_Cl_2_ and allowed to incubate for thirty minutes before evaporating under reduced pressure. Carbon disulfide (0.60 mL, 1,000 equiv.) and then propylene oxide (0.35 mL, 500 equiv.) were added and stirred at 23°C for 5 h. An aliquot was removed for crude NMR analysis of conversion based on remaining propylene oxide and the remainder of the system was quenched and precipitated with 5% HCl/MeOH, to yield a red viscous polymer and red MeOH layer. The samples were centrifuged at four thousand rpm for 5 minutes to separate the viscous and liquid components. The viscous layer was redissolved in CH_2_Cl_2_ and reprecipitated with acidic MeOH a total of three times until the MeOH layer was no longer colored yielded a yellow polymer precipitate. The tacky material was collected, dried under vacuum, and analyzed by GPC, DSC, and NMR and taken as the polymer mass, while the methanol layers were concentrated for analysis of the cyclic byproducts. ^1^H NMR (500 MHz, CDCl_3_) δ = 5.94 (m, CH dithiocarbonate C=S), 5.60 (br s, CH monothiocarbonate C=S), 5.25 (m, CH monothiocarbonate C=S), 5.01 (br s, CH carbonate), 3.84–3.35 (m, CH_2_ and CH trithiocarbonate), 1.45–1.40 (m, CH_3_) ppm. ^13^C NMR (125 MHz, CDCl_3_) δ = 222.27 (trithiocarbonate), 212.49 (dithiocarbonate C=S), 193.21 (monothiocarbonate C=S), 169.66 (monothiocarbonate C=O), 153.43 (carbonate), 41.06 (CH_2_), 18.80 (CH_3_) ppm. IR (ATR): 2,965, 2,909, 1740, 1716, 1,647, 1,449, 1,377, 1,348, 1,259, 1,223, 1,013, and 797 cm^−1^.

CHO/CS_2_ copolymerization: a 25 mL Schlenk flask was dried in the oven overnight, brought into glovebox, and catalyst (0.01 mmol, 1.0 equiv.) and bis (triphenylphosphine) iminium chloride (0.01 mmol, 1.0 equiv.) were added. The solids were dissolved in 2 mL of CH_2_Cl_2_ and allowed to incubate for thirty minutes before evaporating under reduced pressure. Carbon disulfide (2.42 mL, 4,000 equiv.) and then cyclohexene oxide (2.02 mL, 2000 equiv.) were added and heated at 50°C for 4 h. An aliquot was removed for crude NMR analysis of conversion based on remaining CHO and the remainder of the system was quenched and precipitated with 5% HCl/MeOH, to yield a red viscous polymer and red MeOH layer. The samples were centrifuged at four thousand rpm for 5 minutes to separate the viscous and liquid components. The viscous layer was redissolved in CH_2_Cl_2_ and reprecipitated with acidic MeOH a total of three times until the MeOH layer was no longer colored yielded a yellow polymer precipitate. The powdery solids were collected, dried under vacuum, and analyzed by GPC, DSC, and NMR and taken as the polymer mass, while the methanol layers were concentrated for analysis of the cyclic byproducts. ^1^H NMR (500 MHz, CDCl_3_) δ = 5.63 (br s, CH), 4.95 (m, CH), 4.71 (br s, CH), 4.31 (m, CH), 3.58 (br s, CH), 3.44 (br s, CH), 2.27–2.00 (m, 2CH_2_), 1.76–1.27 (m, 2CH_2_) ppm. ^13^C NMR (125 MHz, CDCl_3_) δ = 219.90 (trithiocarbonate), 168.63 (monothiocarbonate C=O), 153.57 (carbonate), 52.06, 47.29, 30.59, 24.44, 22.86 ppm. IR (ATR): 2,936, 2,857, 1743, 1702, 1,448, 1,243, 1,138, 1,065, 1,000, and 936 cm^−1^.

### 2.3 Ellipsometry

Polymer samples were dissolved in CH_2_Cl_2_ (1 g/mL) and spun-coated onto Piranha-cleaned silicon wafers (*ca.* 2 cm × 2 cm). The samples were annealed in a vacuum oven above the samples *T*_g_ overnight.

Two steps were taken to implement a reliable analysis of the ellipsometer data. First, we directly measured thickness of the spun-coated films using atomic force microscopy (AFM; Bruker Dimension Icon) in the tapping-mode. The spun-coated films were scored with a sharp razor blade to create a distinct edge, that allowed procurement of thickness profiles. The directly measured thickness eliminated the term as a fitting parameter in modeling the ellipsometric parameters.

Second, depending on the optical characteristics of the sample, if the sample does not absorb energy (imaginary part of the complex refractive index, *i.e.*, *κ* ≈ 0) then the Cauchy equation can be used to use to determine the real part of the complex refractive index (*n*) profile of the material (Equation 1) (Pavunny et al., 2012).

$$n\left(\lambda \right)=A+\frac{B}{\lambda^{2}}+\frac{C}{\lambda^{4}},$$ (1)

where *n* is the real part of complex refractive index, *λ* is wavelength in *nm*, and *A,B,C …* , are fitting coefficients. Additionally, if the sample does absorb light, the Cauchy–Urbach dispersion model can be used which incorporates an equation to calculated *κ* (Equation 2) (Pavunny et al., 2012).

$$\kappa \left(\lambda \right)=a\;e^{\beta \left(\frac{1239.84}{\lambda }-E_{b}\right)},$$ (2)

where *κ* is imaginary part of the complex refractive index, *α* and *β* are constants, *λ* is wavelength in *nm*, and *E*_*b*_ is the band gap energy in *eV*. Thus, choosing the critical Kramers–Kronig consistent dispersion model is essential in modeling the ellipsometric parameters to calculate the complex refractive index (n*) dispersion.

The ellipsometric parameters [delta (Δ) and psi (ψ)] of the thin films were collected at 23°C via a Nulling Ellipsometry method at varying angles of incidences (50°, 55°, and 60°) across a spectroscopic wavelength from 360 nm to 1700 nm using an Imaging Ellipsometer (Nanofilm EP4; Accurion GmbH, Germany). The parameters of the n* dispersion model used in the fitting of the ellipsometric parameters are presented in Supplementary Table S1. The Δ and Ψ curves, with the appropriate thickness values and n* dispersion model, were modeled in the commercial EP4Model data analysis software by Accurion GmbH, Germany.

## 3 Results and discussion

### 3.1 Epoxide/CS_2_ copolymerization

The effect of the active metal center was investigated by synthesizing chromium, cobalt, and aluminum phenylene diamine salens (**1**, **2**, and **3**, respectively) (Ren et al., 2009; Hosseini Nejad et al., 2012). The copolymerization of epoxides (PO and CHO) with CS_2_ was investigated with these various catalysts using bis (triphenylphosphine) iminium chloride (PPNCl) as a nucleophilic co-catalyst initiator (Table 1). When PO/CS_2_ copolymerizations were conducted in CH_2_Cl_2_ using equimolar PPNCl, only the chromium systems resulted in epoxide conversion. With the phenylene diamine Cr salen (**1**) no catalytic activity was observed in the CHO/CS_2_ reaction, but PO/CS_2_ copolymerization (Entry 1) yielded low molar mass polypropylene thiocarbonates (*M*_n_ = 3,200 g/mol).

**TABLE 1:** Polymerization results, thermal properties, and sulfur content of the resulting products. *^a^*

Entry (#)	Cat	Epox	Conv. (%)^b^	Cyclic (%)^b^	TON^c^	*M*_n_ (g/mol)^d^	*Đ* ^d^	*T*_g_ (°C)^e^	*T*_d_ (°C)^f^	Sulfur (wt%)^g^
1	**1**	PO	93	43	496	3,200	1.74	9	148	46
2	**1**	CHO	0	-	-	-	-	-	-	-
3	**5**	PO	10	71	57	2,400	1.44	−23	131	45
4	**5**	CHO	0	-	-	-	-	-	-	-
5	**6**	PO	54	94	353	1,500	1.77	−3	153	44
6	**6**	CHO	>99	>99	2009	-	-	-	-	-
7	**7**	PO	>99	60	519	3,000	1.70	−4	114	45
8	**7**	CHO	>99	88	1901	3,400	1.67	52	96	29
9	**8**	PO	91	61	880	2,800	1.98	8	130	46
10	**8**	CHO	69	70	190	4,000	1.57	93	181	32
11	**2**	PO or CHO	0	-	-	-	-	-	-	-
12	**3**	PO or CHO	0	-	-	-	-	-	-	-
13	**4**	PO or CHO	0	-	-	-	-	-	-	-
14	**9**	PO or CHO	0	-	-	-	-	-	-	-

^a^ Cat = catalyst from Figure 1. Conditions and reagents: PO:CS_2_:cat = 500:1,000:1, 23°C, 5 h, [catalyst] = 5 mM in CH_2_Cl_2_, quenched in 5% HCl/MeOH; CHO:CS_2_:cat = 2000:4,000:1, 50°C, 4 h, [catalyst] = 5 mM in CH_2_Cl_2_, quenched in 5% HCl/MeOH.

^b^ Determined by mass of polymer precipitate vs. soluble cyclic extracts.

^c^ Determined according to moles of epoxide converted/moles of catalyst.

^d^ Measured by size exclusion chromatography using THF, eluent and PS, calibrated columns.

^e^ Measured from the second heating cycle inflection point of DSC, at 10° per minute.

^f^ Measured at 95 wt% remaining according to thermogravimetric analysis.

^g^ Determined from molar mass ratios of repeat units observed in integrated 13C NMR, spectra.

When the catalyst backbone was changed to non-planar geometries, as in the *trans*-cyclohexadiamine (**7**) and ethylene diamine (**8**) salen ligands, the Cr catalysts showed polymerization activity in both PO and CHO/CS_2_ copolymerizations. By GPC/SEC analysis, CHO/CS_2_ polymers were consistently higher molar mass than PO/CS_2_ products synthesized from the same catalyst (entries 7, 8 & entries 9, 10). We hypothesize this reactivity difference arises from the ability for PO to react through axial binding to the metal center, whereas CHO requires a cis-ligand geometry (e.g., pseudo-octahedral) to undergo the O-S exchange reaction and/or propagation; similar to what has been observed in epoxide/CO_2_ copolymerization by porphyrin catalysts (Chatterjee and Chisholm, 2013). This cis-ligand geometry is less accessible in the planar phenylene diamine backbone (**1**) and thus only PO catalysis was productive. Whereas both Co. and Cr catalysts are active in epoxide/CO_2_ copolymerization and Al catalysts are active in epoxide/anhydride copolymerization (Longo et al., 2016), only Cr catalysts were active in epoxide/CS_2_ copolymerization. It is expected that the combined Lewis acidity, oxophilicity, and thiophilicity of Cr are key thermodynamic considerations for promoting catalyst activity in both propagation and O-S exchange mechanisms (Kepp, 2016; Zhang et al., 2020).

The electronics and steric environments of Cr (salph) systems (**4**–**6**) were next investigated. Unsubstituted salicylaldehyde derived catalyst (**4**) exhibited no CS_2_ copolymerization with PO or CHO, which we attribute to poor catalyst solubility. Electronic deficient para-fluoro (**5**) and electron rich para-methoxy (**6**) catalysts revealed that electron rich systems were more reactive and produced higher molar masses by GPC (2,400 and 1,500 g/mol, respectively). The electronics enhance nucleophilicity of the dithiocarbonate chain-end (Darensbou et al., 2013), and accelerates the cyclization reaction and increases the observed yields of cyclic over polymer (entries 5 and 6). Dinuclear tethered cocatalyst system **9** (Ren et al., 2009), in the absence or presence of 1 equiv. of PPNCl failed to afford any conversion, consistent with the other cobalt catalysts studied.

**FIGURE 2:**
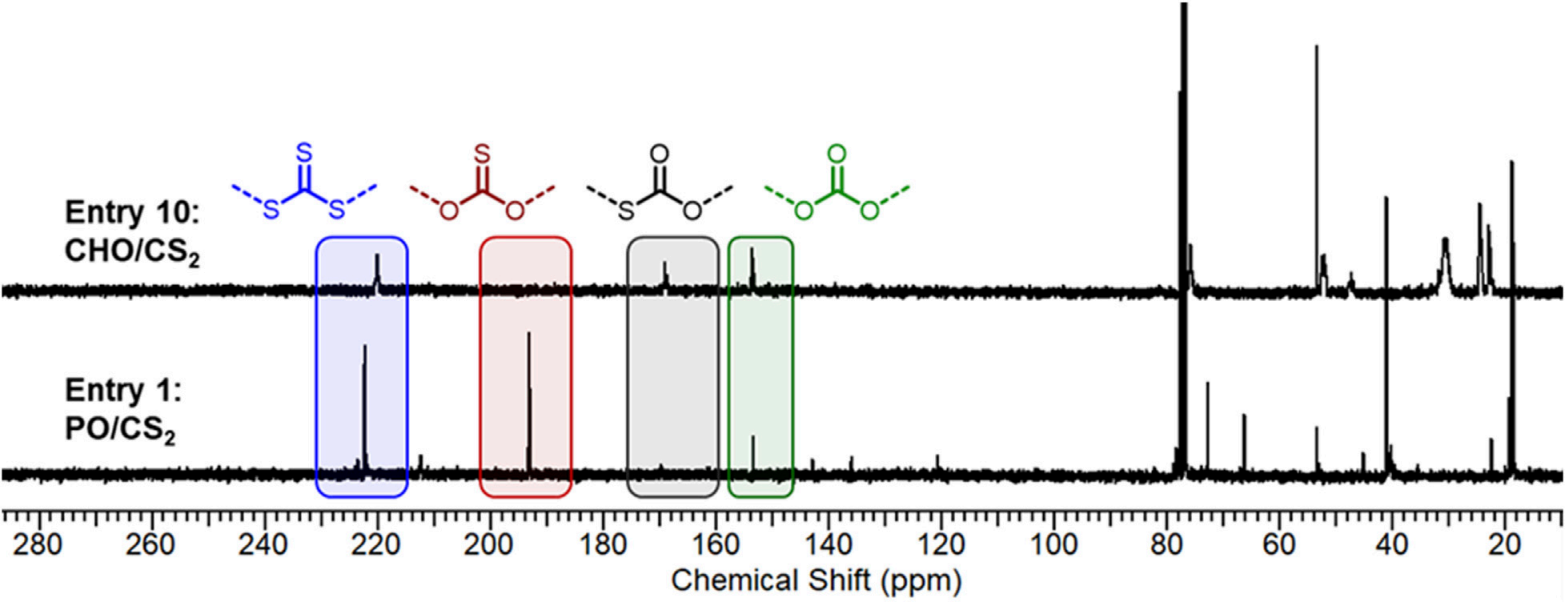
^13^C NMR analysis (125 MHz, CDCl_3_) and comparison of microstructure distribution in PO (Entry 1) and CHO (Entry 10) copolymers.

The obtained O-S exchange microstructures in productive catalyst systems were investigated by ^13^C NMR to ascertain the sulfur content (wt%) and ratio of trithiocarbonate, mono thiocarbonate (C=O, C=S), and carbonate linkages (Figure 2). While there are six possible combinations (Diebler et al., 2016) of exchange repeat units—S (C=S)S, S (C=S)O, S (C=O)S, O (C=S)O, S (C=O)O, O (C=O)O—four prominent microstructures were produced by Cr salen catalysts. The NMR of entry 1 (Figure 2; Supplementary Figures S1, S2) exhibited predominantly trithiocarbonate resonances (43 mol%) at chemical shifts of 222 ppm and monothiocarbonates (C=S, 40 mol%) at 193 ppm. The remaining linkages were non-sulfur containing carbonate linkages (9 mol%) and trace amounts of dithio- and monothiocarbonates (C=O). This indicates that under metal-catalyzed conditions, O-S exchange can produce polymeric materials with higher sulfur content than the unexchanged dithiocarbonate products.

Whereas catalyst electronics and ligand geometry dictated monomer selectivity and polymerization productivity, there were only minor differences (within 5 mol% deviations by ^13^C NMR) observed in the microstructures within a given epoxide study (Supplemental Figures S1–S10). However, we did observe significant differences in microstructures between CHO and PO. Compared to the above PO/CS_2_ polymer (Figure 2), CHO/CS_2_ copolymerization afforded predominantly trithiocarbonate (52%) and carbonate linkages (31%) with a minor monothiocarbonate (C=O) component (16%). Interestingly, the differences in monothiocarbonate isomers (C=S with PO and C=O with CHO) points to a difference in O-S exchange mechanisms that is dependent on the monomer and catalyst system. In both systems the unexchanged dithiocarbonate (C=S) is observed in only slight amounts (<5 mol%) by ^1^H NMR. Given the observations that propylene oxide/COS copolymers selectively yield monothiocarbonates with C=O moieties rather than C=S, we propose our observed products form from post-polymerization O-S exchange of a carbonate moiety rather than direct incorporation of in-situ generate COS molecules (Luo et al., 2016). Because CHO is more sterically hindered and conformationally restricted, the necessary transition state for this post-polymerization exchange is suppressed and the carbonate moieties are observed (Zhang et al., 2008; Darensbourg et al., 2009; Darensbou et al., 2013; Yang et al., 2020).

**FIGURE 3:**
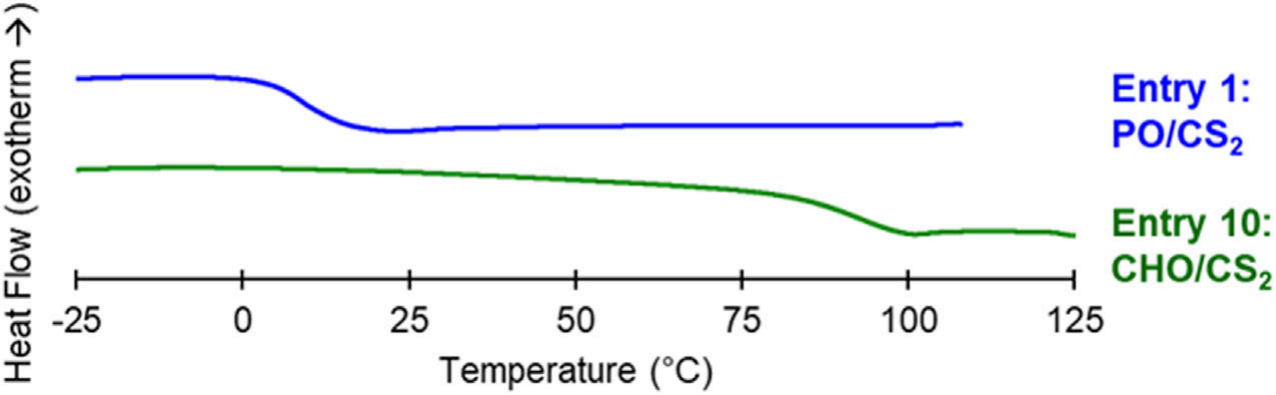
DSC of adhesive PO/CS_2_ (Entry 1) and plastic CHO/CS_2_ (Entry 10) copolymers. Second heat shown at a rate of 10°C min^-1^ and glass transition values taken at the inflection point.

The thermal properties of the CHO/CS_2_ and PO/CS_2_ copolymers were measured by differential scanning calorimetry (DSC) in order to ascertain their potential as optical plastics and adhesives, respectively (Figure 3; Supplemental Figure S11). The cyclohexyl structure restricts molecular mobility throughout the chain and rigidifies the polymers giving rise to high *T*_g_ materials (*T*_g_ = 93°C). The higher *T*_g_ is essential for plastic and rigid optical components. Alternatively, the flexible linear propylene linkage imparts rubbery properties (*T*_g_ = 9°C) after precipitation and purification from cyclic plasticizers. Compared to ethylene oxide/CS_2_ copolymers (Yang et al., 2021), the obtained polymers are entirely amorphous due to the monosubstituted and di-substituted epoxides used which disrupt chain packing in these atactic poly (thio) carbonates. These amorphous PO/CS_2_ copolymer structures exhibit the low *T*_g_s needed for imparting flexibility and peel strengths properties.

### 3.2 Optical properties

**FIGURE 4:**
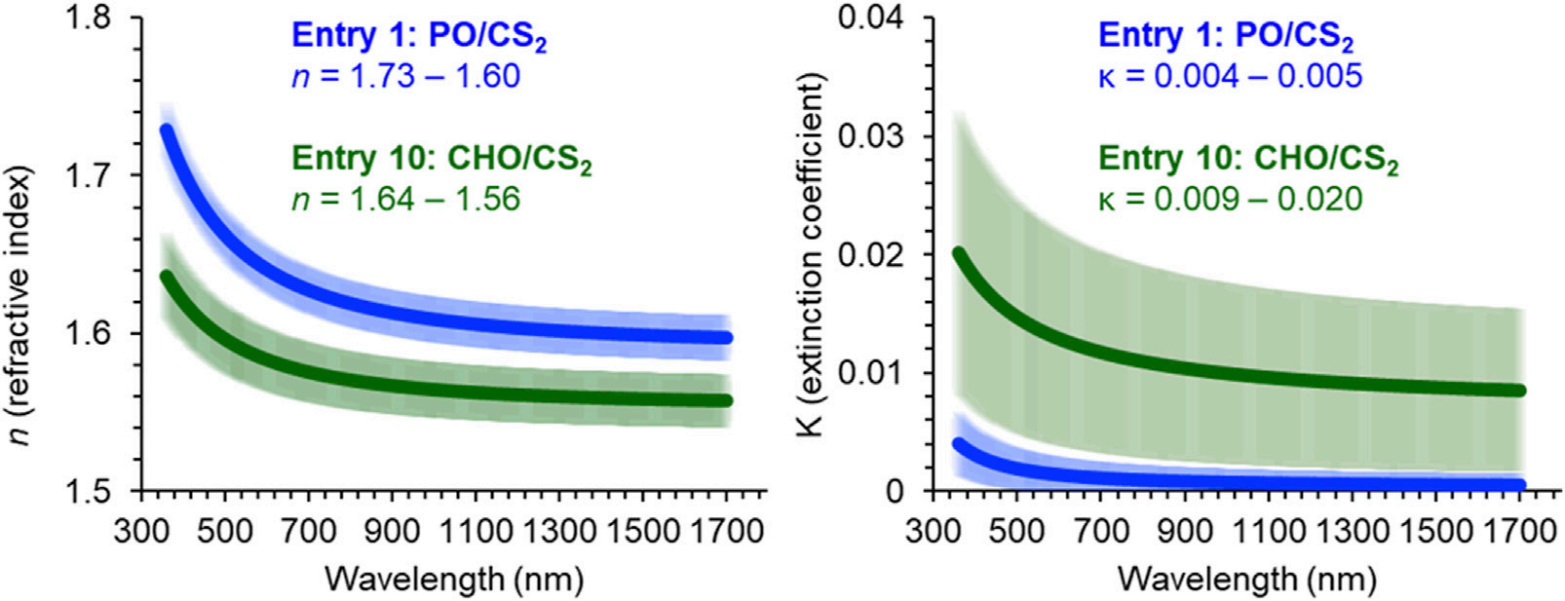
Refractive index and extinction coefficient values across 360–1,700 nm as measured by variable-angle spectroscopic ellipsometer for PO and CHO/CS_2_ copolymers.

Investigation of the optical parameters in the CS_2_/PO or CHO systems was carried out using variable-angle spectroscopic ellipsometry. This optical measurement (Figure 4) provides the *n* and *κ* across wavelengths (λ) in order to ascertain the utility of a material in high refractive index, low-absorbance, multilayer devices. In the PO/CS_2_ sample (entry 1), the range of *n* was 1.73–1.60 (λ = 360–1700 nm), which compares favorably to *n* = 1.78 of episulfide derived CS_2_ poly (trithiocarbonates) (Nakano et al., 2007). Encouraging was the low absorbance extinction coefficient for PO/CS_2_ copolymer (*κ* = 0.004–0.005) across the studied spectrum (λ = 360–1700 nm).

Another characteristic that can be derived from these optical constants is the Abbe number (*V*_D_) which is a measure of dispersion, how the refractive index changes with wavelength, within a transparent material and is defined by equation 4. A higher *V*_D_ corresponds to a low dispersion material, which improves that ability for the material to focus light. Entry 1 exhibits a very high dispersion with a *V*_D_ = 19.1, while the CHO/CS_2_ copolymer (entry 10) exhibits a more favorable Abbe number (*V*_D_ = 32.2), which is comparable to polycarbonate glasses. While the CHO-based materials demonstrates improved dispersive properties, it is accompanied by lower sulfur content (28 wt%), a lower refractive index (*n* = 1.64–1.56), and higher absorbance (*κ* = 0.009–0.020).

$$V_{D}=\frac{n_{d}-1}{n_{F}-n_{C}}$$ (3)

Equation 3, where: *n*_F_ is the refractive index at wavelength of the Fraunhofer F spectral line (486 nm), *n*_d_ is the refractive index at wavelength of the Fraunhofer d spectral line (587 nm) and *n*_C_ is the refractive index at wavelength of the Fraunhofer C spectral line (656 nm).

## 4 Conclusion

**FIGURE 5:**
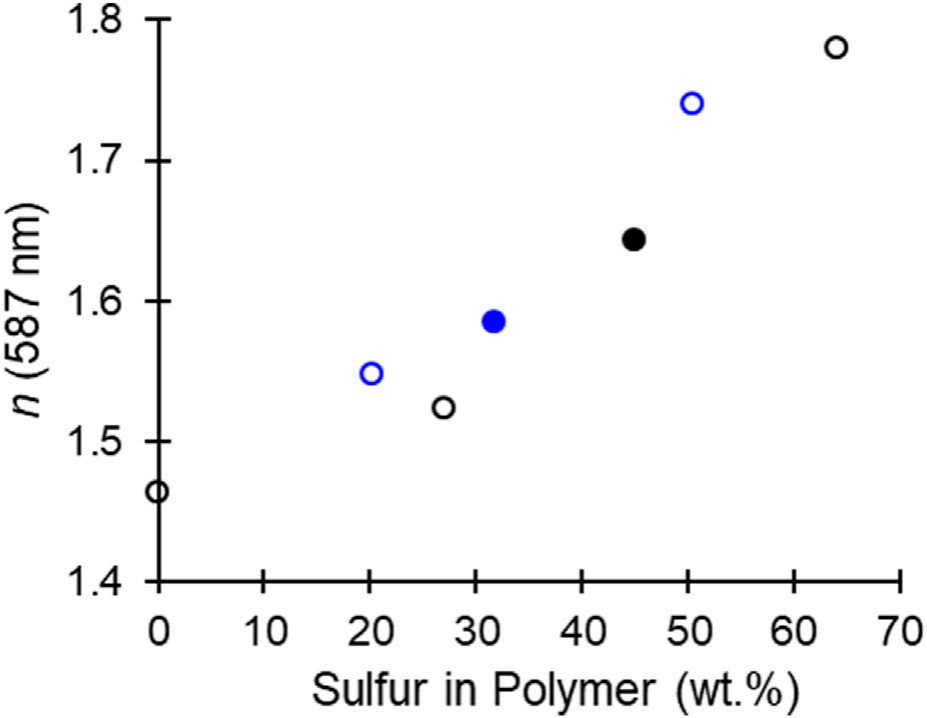
Comparison of refractive index and sulfur content in oxirane/CSX (X = S or O) copolymers (Nakano et al., 2007; Zhang et al., 2008; Darensbourg et al., 2009; Zhang et al., 2009; Yang et al., 2020). Black corresponds to low *T*_g_ propylene materials, blue corresponds to high *T*_g_ cyclohexyl materials, open points are literature values, and closed points are from this study.

The metal catalyst design in epoxide/CS_2_ copolymerization was shown to dictate monomer selectivity and polymerization productivity. By matching the catalyst selection with epoxide choice (CHO or PO), high *T*_g_ plastics (*T*_g_ = 93°C) with high refractive indexes (*n* up to 1.64), modest absorbance (*κ* < 0.020), and good Abbe numbers (*V*_D_ = 32.2) could be synthesized along with low *T*_g_ adhesives (*T*_g_ = 9°C) with high refractive indexes (*n* up to 1.73), very low absorbance (*κ* < 0.005), and low Abbe numbers (*V*_D_ = 19.1). Within a given epoxide monomer the microstructures were consistent, PO/CS_2_ copolymers all produce materials with 45 ± 1 sulfur wt% and CHO/CS_2_ copolymers produce materials with 30 ± 2 sulfur wt%. However, CHO microstructures were dominated by trithiocarbonates and monothiocarbonates with thiocarbonyl moieties (C=S); whereas PO microstructures were dominated by trithiocarbonates and carbonate moieties. In the theoretical polymerization where no O-S exchange occurs PO yields polymers with 48 wt% and CHO 37 wt%, which indicates the metal-catalyzed exchanged decreases the optimal sulfur content for high refractive index. However, by suppressing the formation of carbonate linkages, CHO/CS_2_ copolymers are relatively closer to their optimal sulfur wt%. From these findings, research may be pursued wherein O-S exchange favors trithiocarbonate formation and S content rivaling episulfide/CS_2_ copolymerization (64 and 51 wt% for PO and CHO, respectively) (Nakano et al., 2007; Zhang et al., 2008; Darensbourg et al., 2009; Zhang et al., 2009; Yang et al., 2020). Context for the materials synthesized in this study and in the literature are presented in Figure 5, wherein various combinations of epoxides, episulfides, CS_2_, CO_2_, and COS enable synthetic control over the sulfur content which directly relates to the material refractive index. From this polymer toolset both the thermal and optical properties can be targeted for a given application.

## Data Availability

The original contributions presented in the study are included in the article/Supplementary Material, further inquiries can be directed to the corresponding author.
