# Supplementary material for: Metal-catalyzed copolymerizations of epoxides and carbon disulfide for high-refractive index low absorbance adhesives and plastics
Source: Front Chem. 2023 Nov 2;11:1287528. doi: 10.3389/fchem.2023.1287528 (PMC10652881; doi:10.3389/fchem.2023.1287528)
Supplement: Supplementary file 1 [file DataSheet1.docx]

Supplementary Material

Metal-Catalyzed Copolymerizations of Epoxides and Carbon Disulfide for High-Refractive Index Low Absorbance Adhesives and Plastics

Derek B. Schwarz, Anvay Patil, Saranshu Singla, Ali Dhinojwala, James M. Eagan*

School of Polymer Science and Polymer Engineering, The University of Akron, Akron, OH, USA.

*** Correspondence:**Corresponding Author
eagan@uakron.edu

# Supplementary Data

General Considerations: All polymerizations were carried out under dry nitrogen using an MBraun Unilab glovebox. ^1^H NMR spectra were recorded using a Varian Inova 500 spectrometer and were referenced versus residual non-deuterated solvent shifts (CHCl_3_ δ = 7.26 ppm) (^1^H). ^13^C NMR spectra of polymers were recorded on a Varian Inova (125 MHz) spectrometer and were referenced versus solvent shifts (CDCl_3_ δ = 77.16 ppm) (^13^C). Molar masses (*M*_n_ and *M*_w_) and molecular mass distributions (*Ɖ* = *M*_w_/*M*_n_) were determined by gel permeation chromatography (GPC). Analyses were performed using a Tosoh EcoSEC HLC-8320 GPC RI Detector with two 17393 TSKgel columns (7.8 mm ID x 30 cm, 13 µm) and one 17367-TSKgel Guard Column (7.5 mm ID x 7.5 cm, 13 µm). Inhibitor-free HPLC grade THF was used as the eluent at a flow rate of 1 mL/min at 40 °C. Data were measured relative to polystyrene standards (*Ð* < 1.05) calibrated less than four months prior to data collection. Polymer glass transition temperatures (*T*_g_s) were measured by differential scanning calorimetry using DSC-TA Discovery DSC 250. Analyses were performed in aluminum pans under nitrogen and data were collected from the second heating run at a heating rate of 10 °C/min from -50 to 100 °C or 125 °C and processed with TA TRIOS Software using the inflection point analysis value. Thermal decomposition temperature (*T*_d_) was measured at 95 wt.% remaining by thermal gravimetric analysis (TGA) using a TA Q500 instrument at a heating rate of 10 °C/min in N_2_ atmosphere. Results were processed with TA TRIOS Software. Infrared (IR) spectra were recorded on a Perkin Elmer Frontier spectrometer equipped with an attenuated total reflection (ATR) system.

# Supplementary Figures and Tables

Supplemental ^1^H NMR, ^13^C NMR spectra, DSC, and TGA are provided below for entries 1 and 10, PO/CS_2_ and CHO/CS_2_ copolymers respectively. IR, AFM height scan, and ellipsometry parameter models.

## Supplementary Figures


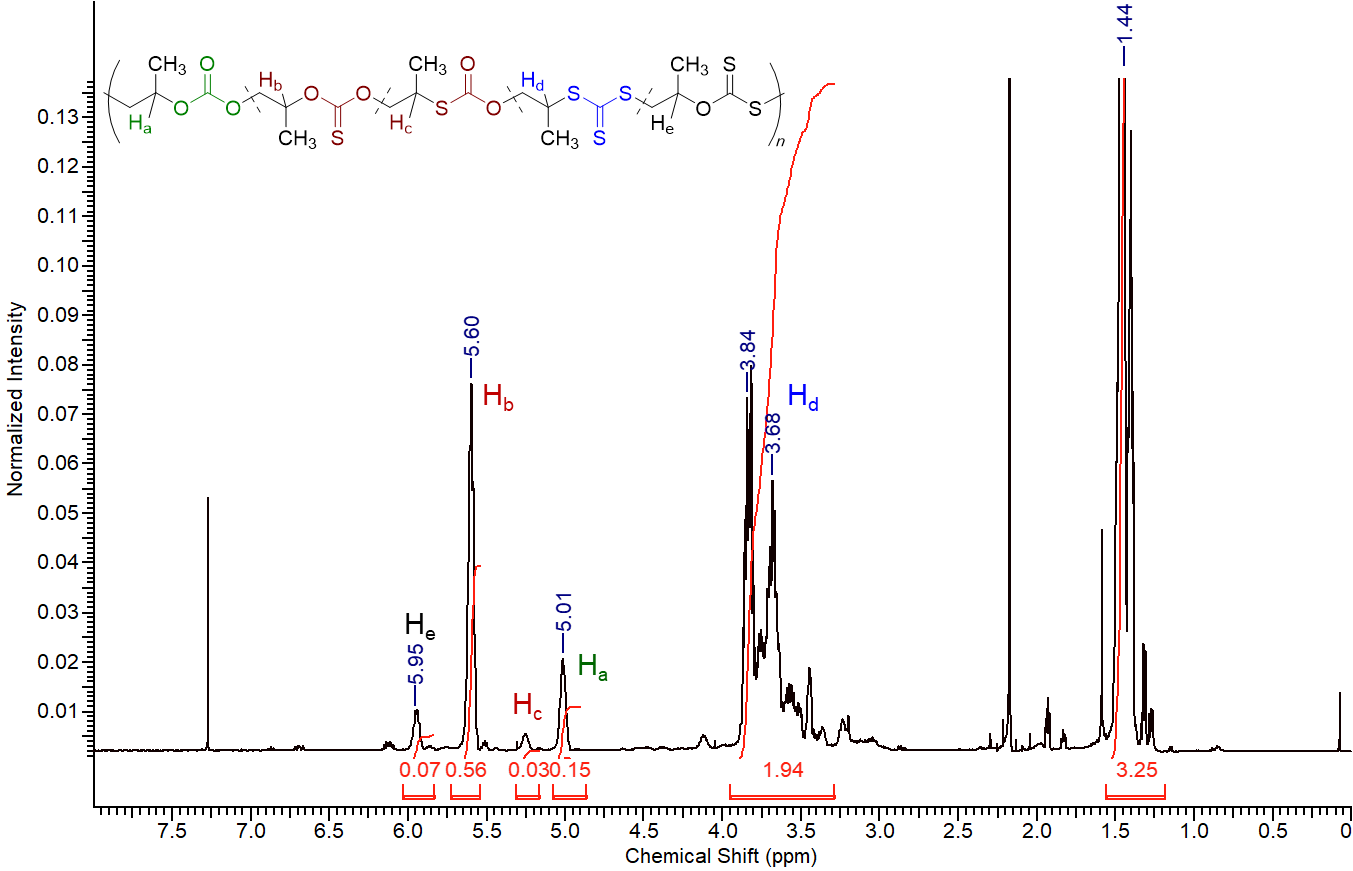


**Supplementary Figure 1.** ^1^H NMR (CDCl_3_) of the copolymer product of PO/CS_2_/**1** (Entry 1).


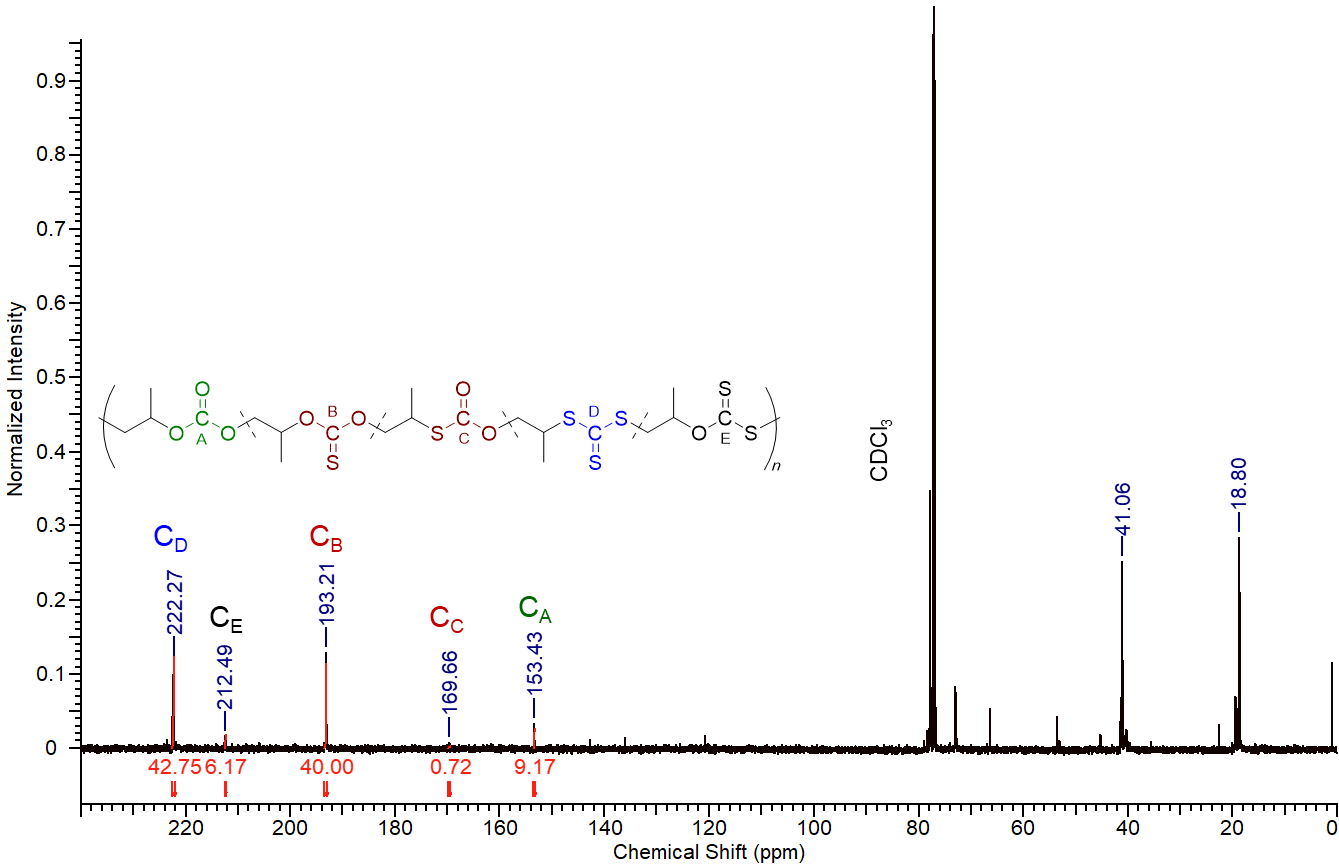


**Supplementary Figure 2.** ^13^C NMR (CDCl_3_) of the copolymer product of PO/CS_2_/**1** (Entry 1).


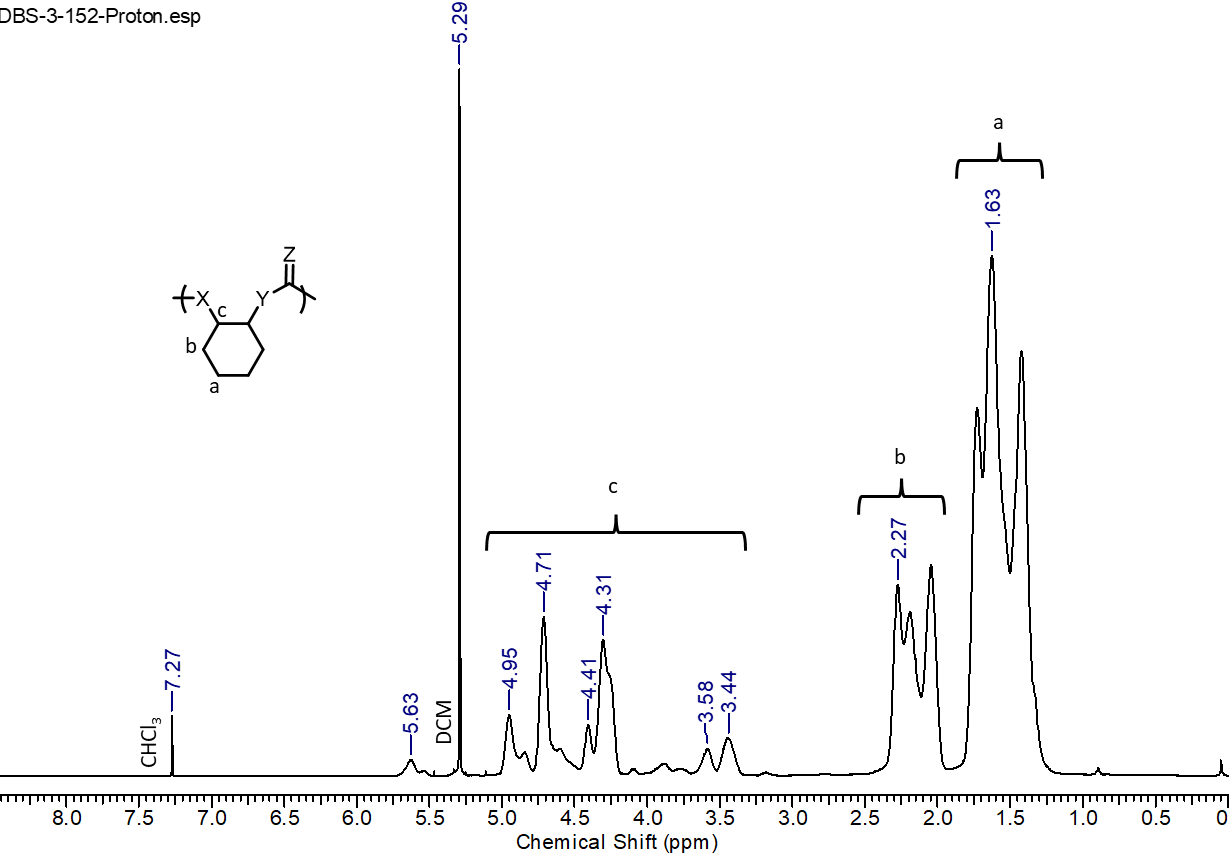


**Supplementary Figure 3.** ^1^H NMR (CDCl_3_) of the copolymer product of CHO/CS_2_/**8** (Entry 10).


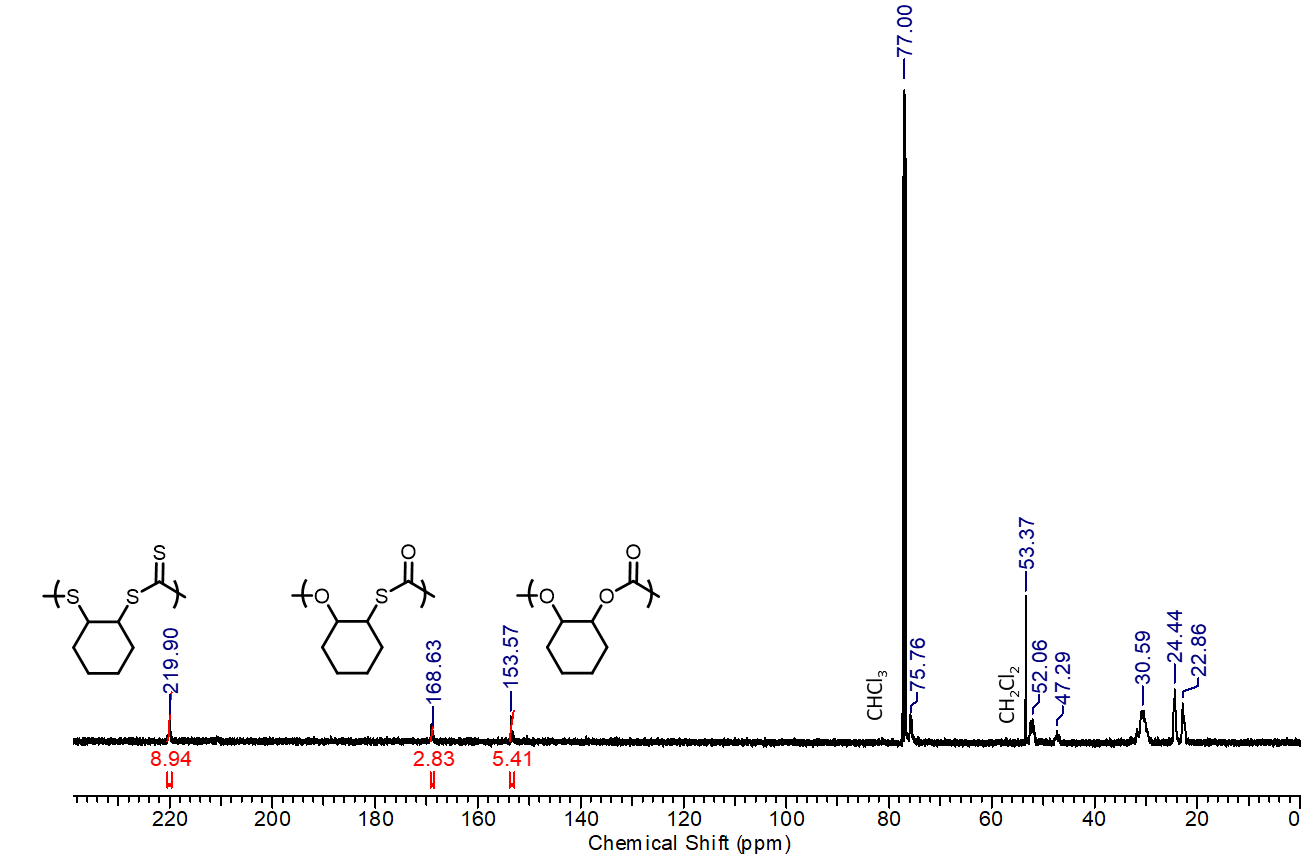


**Supplementary Figure 4.** ^13^C NMR (CDCl_3_) of the copolymer product of CHO/CS_2_/**8** (Entry 10).

**
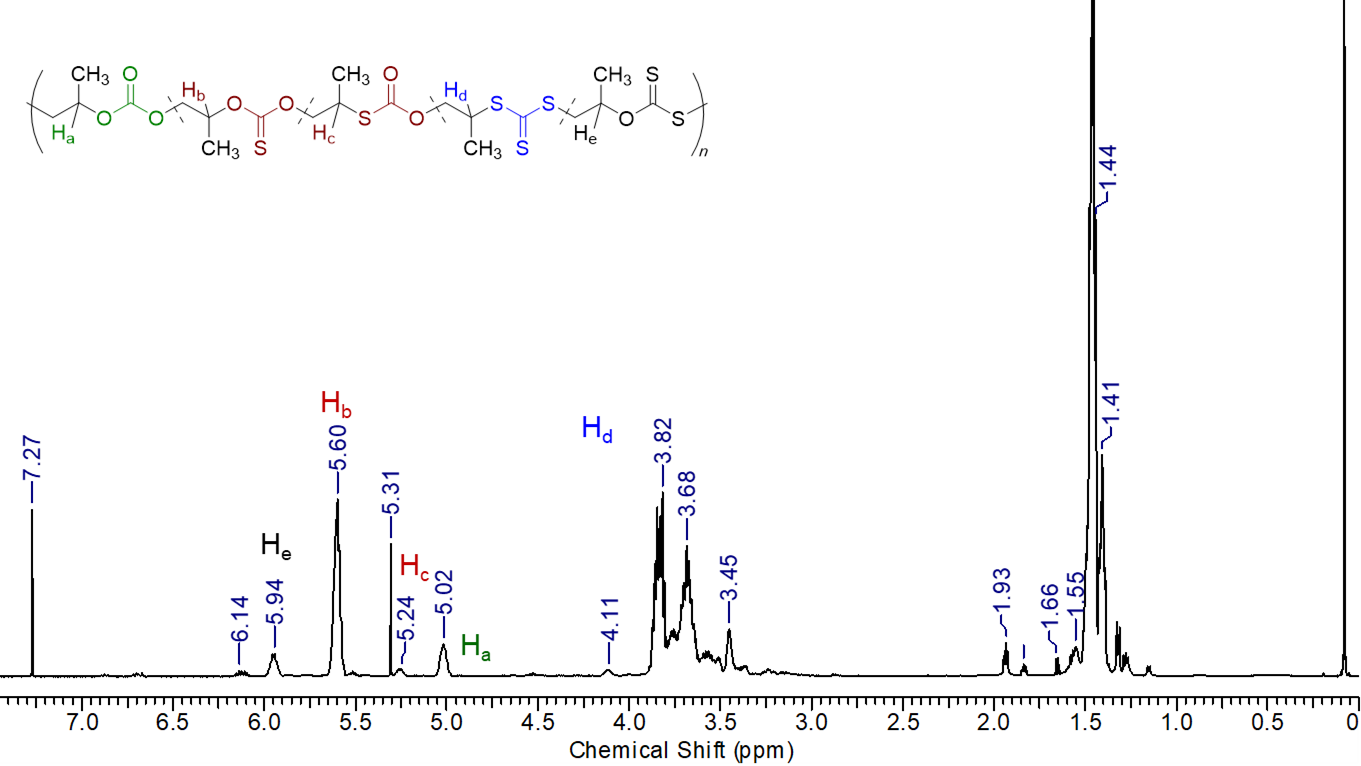
**

**Supplementary Figure 5.** ^1^H NMR (CDCl_3_) of the copolymer product of PO/CS_2_/**5** (Entry 3).


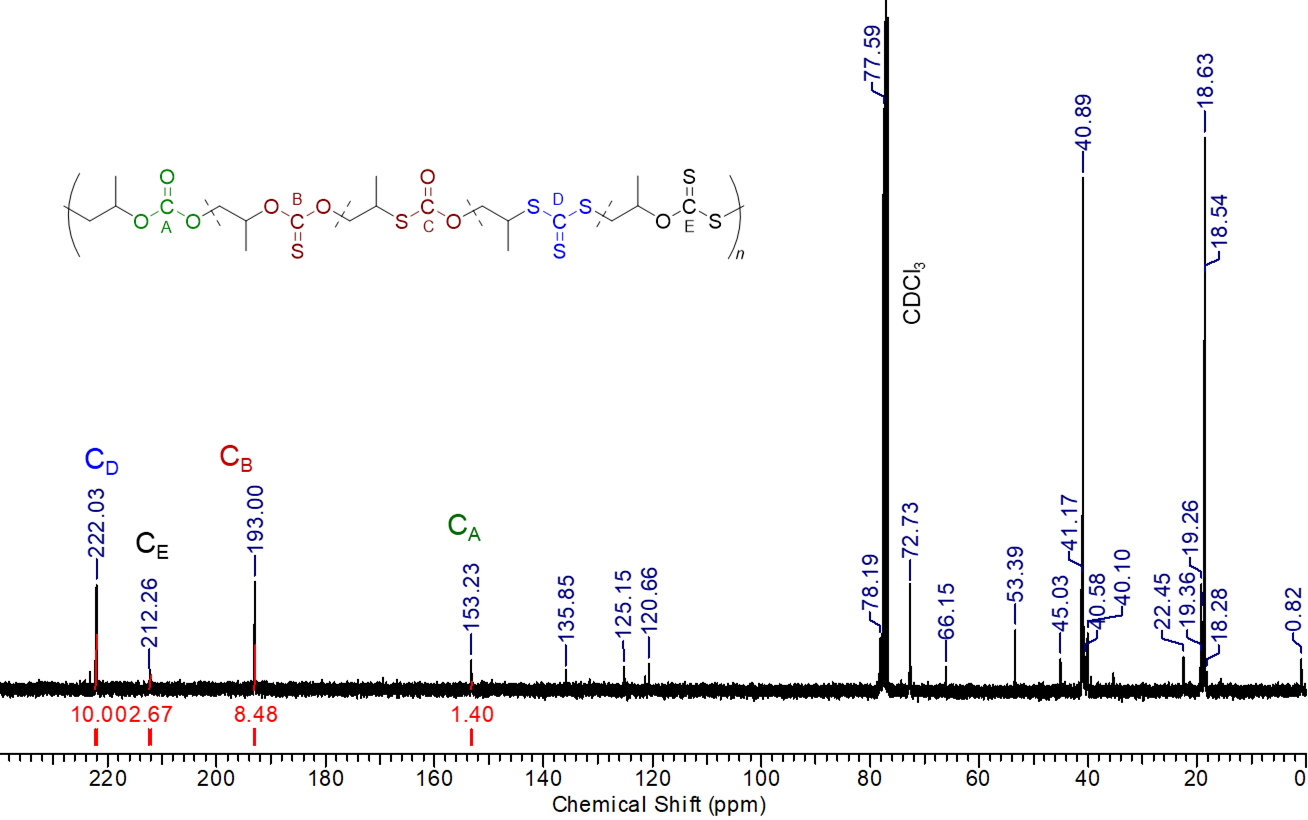


**Supplementary Figure 6.** ^13^C NMR (CDCl_3_) of the copolymer product of PO/CS_2_/**5** (Entry 3).

**
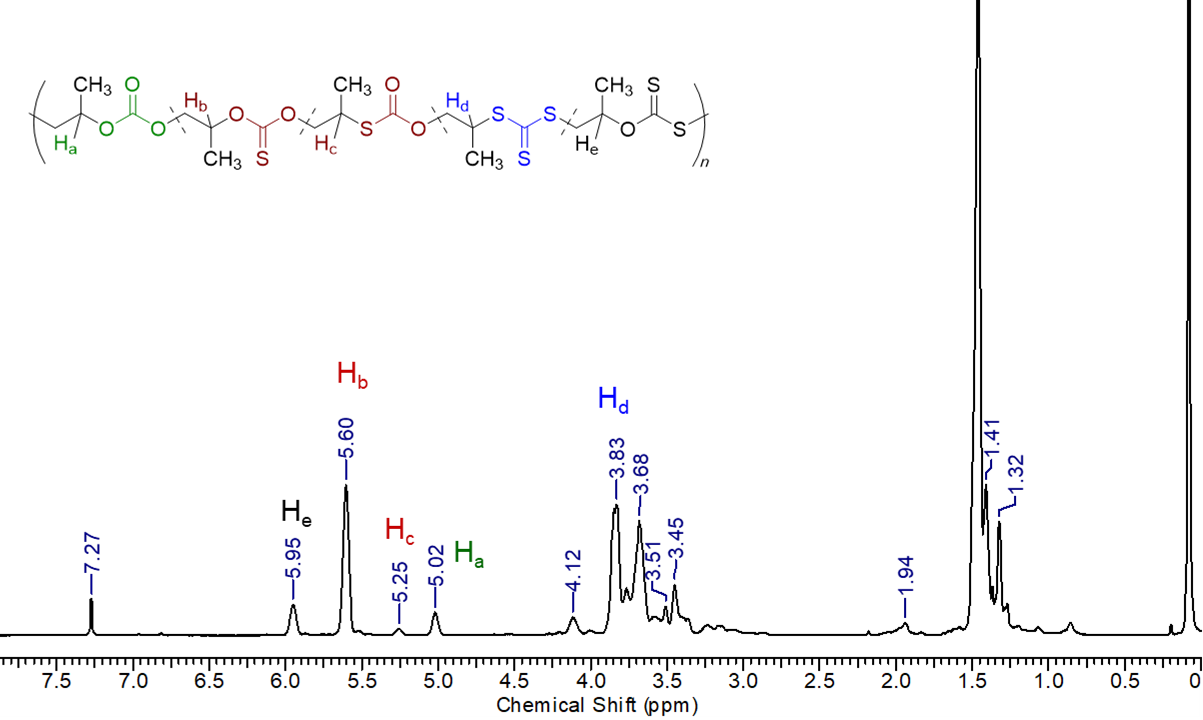
**

**Supplementary Figure 7.** ^1^H NMR (CDCl_3_) of the copolymer product of PO/CS_2_/**6** (Entry 5).


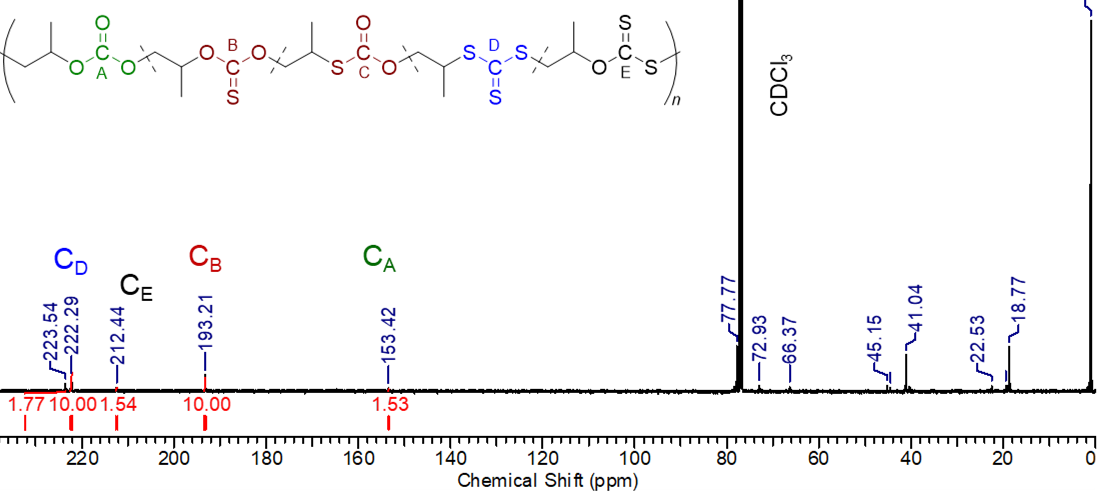


**Supplementary Figure 8.** ^13^C NMR (CDCl_3_) of the copolymer product of PO/CS_2_/**6** (Entry 5).

**
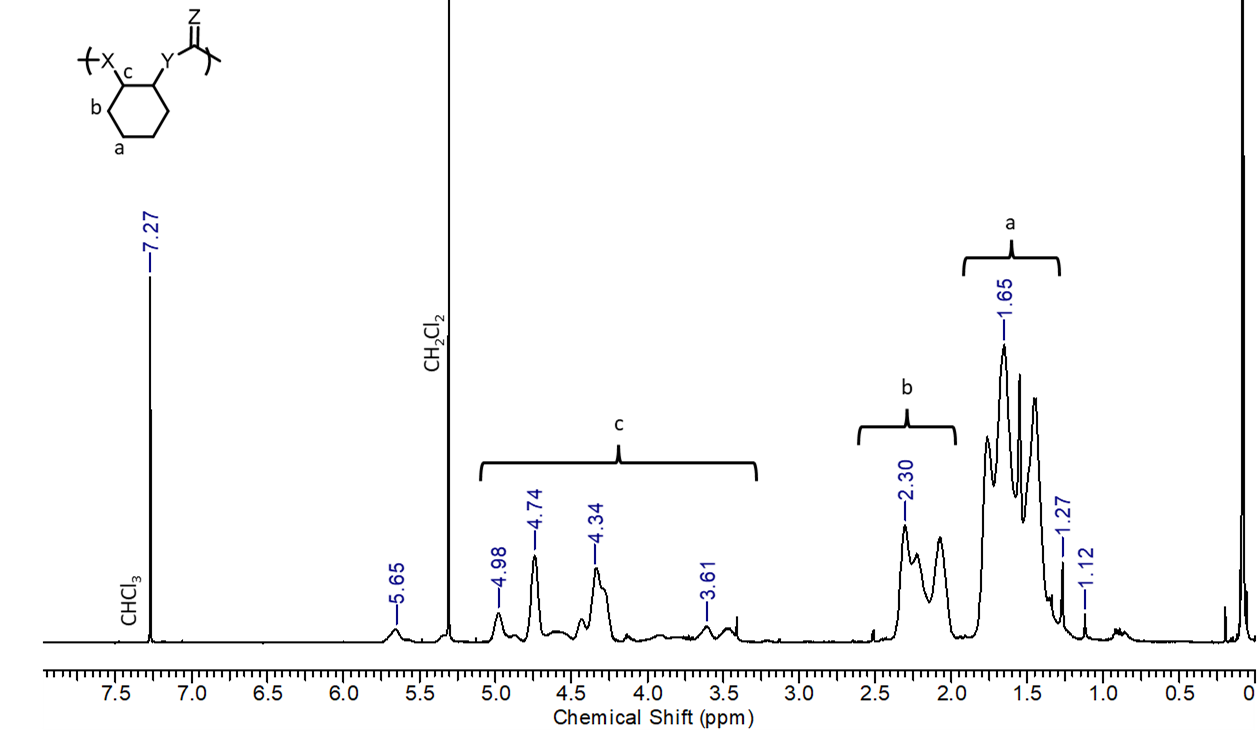
Supplementary Figure 9.** ^1^H NMR (CDCl_3_) of the copolymer product of CHO/CS_2_/**7** (Entry 8).

**
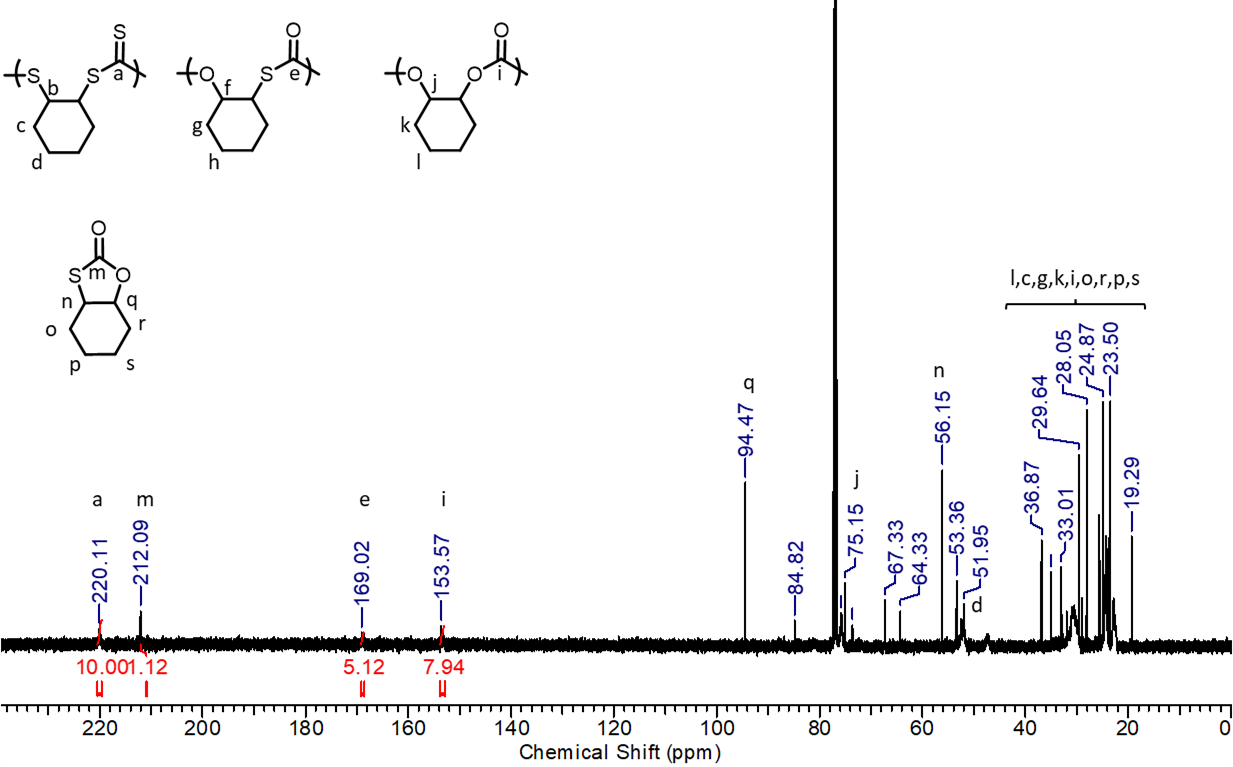
Supplementary Figure 10.** ^13^C NMR (CDCl_3_) of the copolymer product of CHO/CS_2_/**7** (Entry 8).


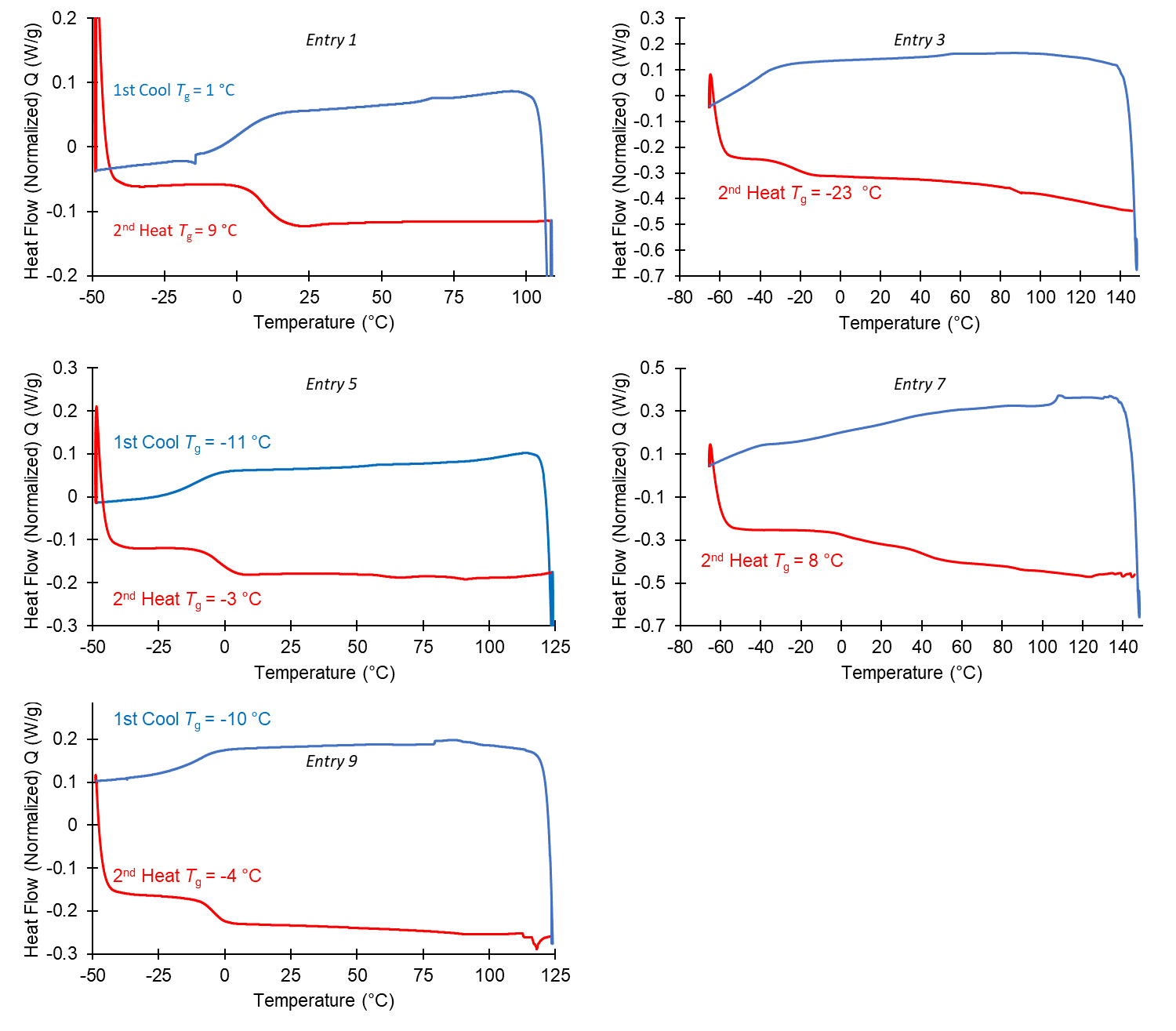
**Supplementary Figure 11.** DSC of the copolymer products of PO/CS_2_ copolymerization (Entries 1, 3, 5, 7, and 9). First cool and second heats shown with exotherms up at a heating rate of 10 °C min^-1^.


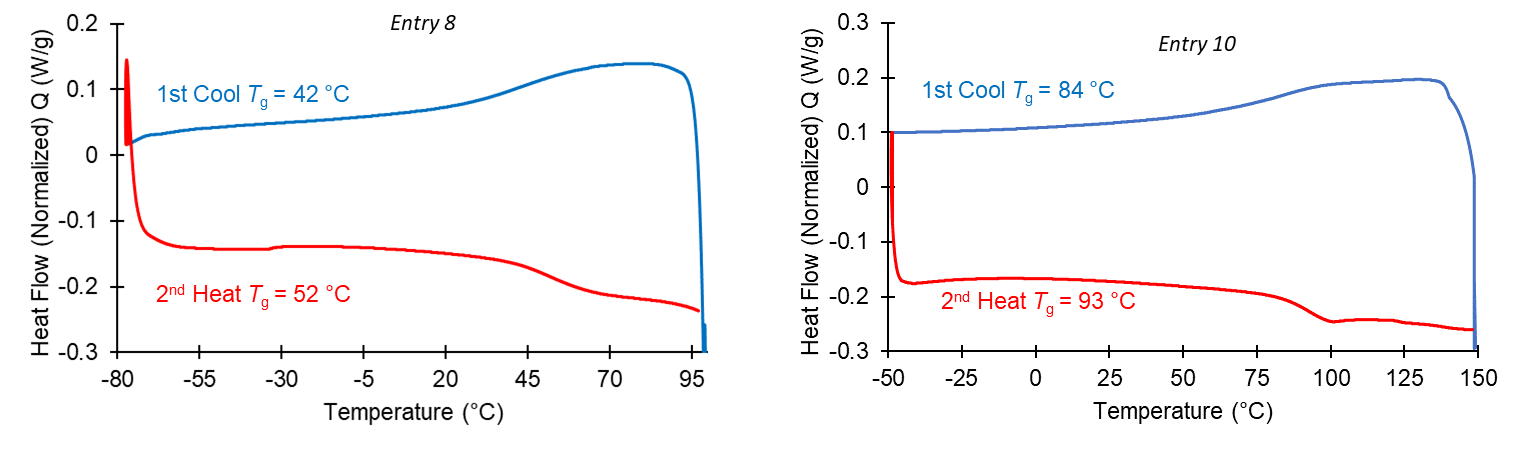
**Supplementary Figure 12.** DSC of the copolymer products of CHO/CS_2_ copolymerization (Entries 8 and 10). First cool and second heats shown with exotherms up at a heating rate of 10 °C min^-1^.


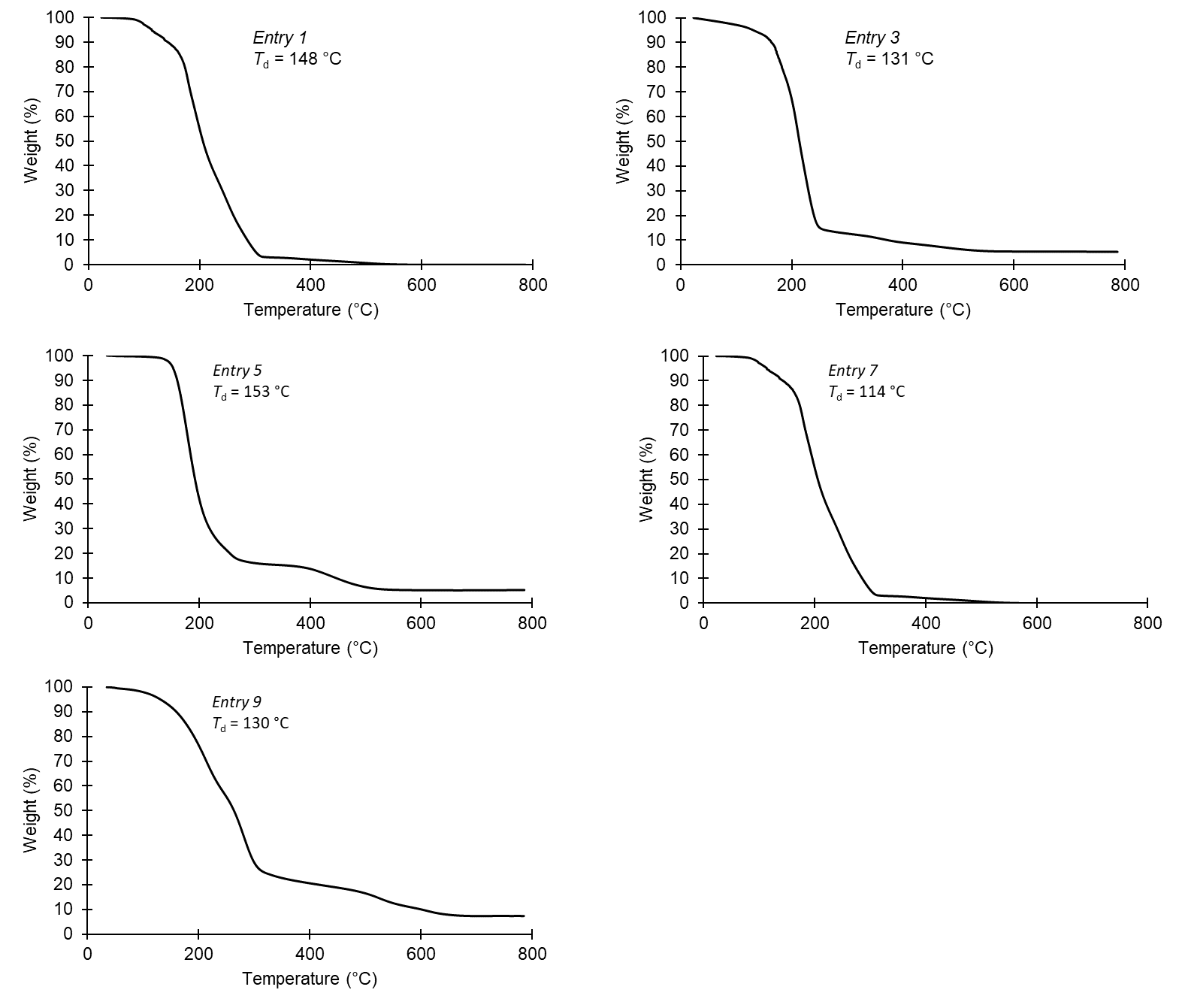
**Supplementary Figure 13.** TGA PO/CS_2_ copolymer products (Entries 1, 3, 5, 7, and 9). Thermal decomposition temperature (*T*_d_) was measured at 95 wt.% remaining.


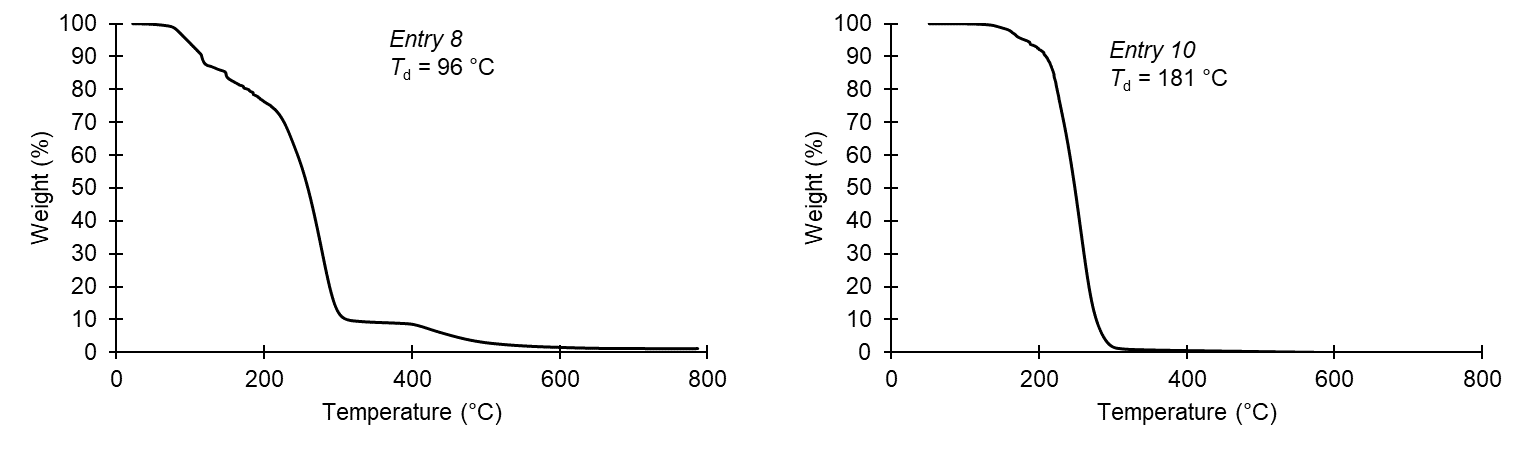
**Supplementary Figure 14.** TGA CHO/CS_2_ copolymers (Entries 8 and 10). Thermal decomposition temperature (*T*_d_) was measured at 95 wt.% remaining.

**Supplementary Figure 15.** Size exclusion chromatography of epoxide/CS_2_ copolymer Entry 1, after precipitation in MeOH. Eluted in THF against PS references using an RI detector.

**Supplementary Figure 16.** Size exclusion chromatography of epoxide/CS_2_ copolymer Entry 3, after precipitation in MeOH. Eluted in THF against PS references using an RI detector.

**Supplementary Figure 17.** Size exclusion chromatography of epoxide/CS_2_ copolymer Entry 5, after precipitation in MeOH. Eluted in THF against PS references using an RI detector.

**Supplementary Figure 18.** Size exclusion chromatography of epoxide/CS_2_ copolymer Entry 7, after precipitation in MeOH. Eluted in THF against PS references using an RI detector.

**Supplementary Figure 19.** Size exclusion chromatography of epoxide/CS_2_ copolymer Entry 8, after precipitation in MeOH. Eluted in THF against PS references using an RI detector.

**Supplementary Figure 20.** Size exclusion chromatography of epoxide/CS_2_ copolymer Entry 9, after precipitation in MeOH. Eluted in THF against PS references using an RI detector.

**Supplementary Figure 21.** Size exclusion chromatography of epoxide/CS_2_ copolymer Entry 10, after precipitation in MeOH. Eluted in THF against PS references using an RI detector.


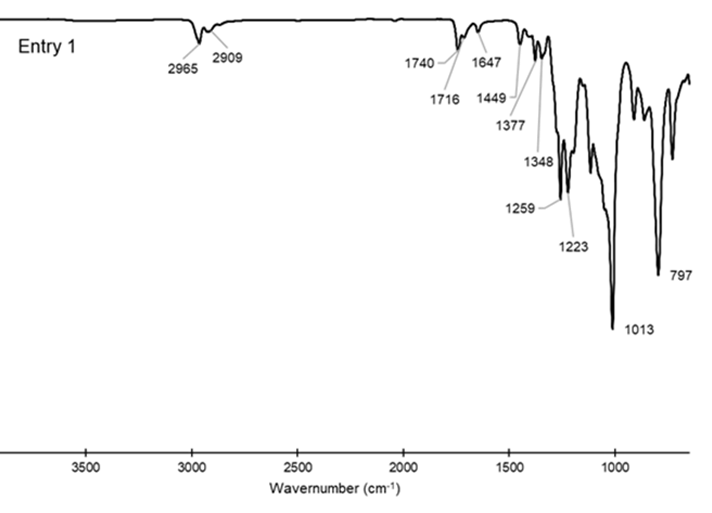


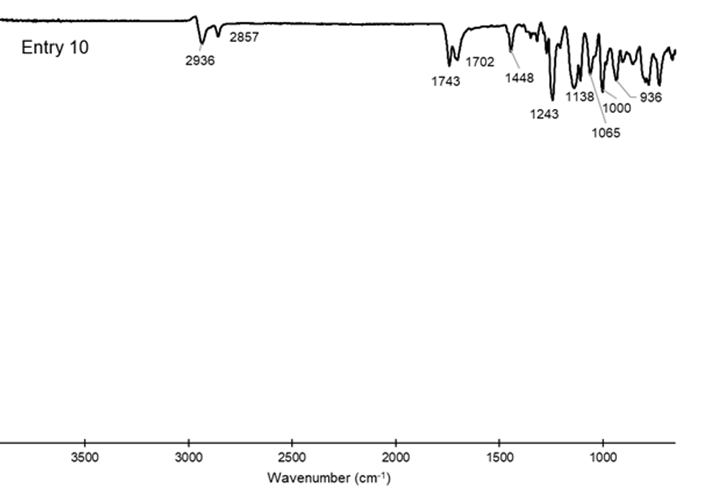


**Supplementary Figure 22.** Infrared spectra of PO/CS_2_ copolymer (entry 1) and CHO/CS_2_ copolymer (entry 10) using attenuated total reflection (ATR).

**Supplementary Table 1. Parameters in the complex *Cauchy-Urbach* dispersion model, used in the fitting of spectroscopic, ellipsometric parameters - Δ and Ψ.**

**a.** Entry 1: PO/CS_2_

|  | *Real Part* | | | | | *Imaginary Part* | | | | *Silica* | | | *PO/CS_2_* |
| --- | --- | --- | --- | --- | --- | --- | --- | --- | --- | --- | --- | --- | --- |
| No. | | *A* | *B* | *C* | *α* | | *β* | *E_b_* | | | *d^a^* | | *D^a^* |
| 1 | | 1.590 | 18544 | 0 | 0.100 | | 0.678 | 8.356 | | | 2.0 | | 315.3 |
| 2 | | 1.597 | 18690 | 0 | 0.100 | | 0.807 | 7.843 | | | 2.0 | | 315.8 |
| 3 | | 1.584 | 17603 | 0 | 0.100 | | 0.746 | 7.573 | | | 2.0 | | 322.2 |
| 4 | | 1.568 | 16980 | 0 | 0.100 | | 0.663 | 6.991 | | | 2.0 | | 327.7 |
| 5 | | 1.602 | 18483 | 0 | 0.100 | | 1.159 | 6.493 | | | 2.0 | | 315.7 |
| 6 | | 1.593 | 18085 | 0 | 0.100 | | 0.506 | 10.585 | | | 2.0 | | 318.6 |
| 7 | | 1.590 | 18120 | 0 | 0.100 | | 0.870 | 7.151 | | | 2.0 | | 316.5 |
| 8 | | 1.596 | 17367 | 0 | 0.100 | | 1.033 | 6.880 | | | 2.0 | | 320.8 |
| 9 | | 1.598 | 16961 | 0 | 0.100 | | 0.897 | 7.274 | | | 2.0 | | 327.0 |
| Avg. | | 1.591 | 17870 | 0.000 | 0.10 | | 0.818 | 7.683 | | | |  |  |
| Std. dev. | | 0.010 | 666 |  |  | | 0.199 | | 1.220 | | |  |  |

*^a^d, D* is thickness in *nm*.

**b.** Entry 10: CHO/CS_2_

|  | *Real Part* | | | | | *Imaginary Part* | | | | *Silica* | | | *CHO/CS_2_* |
| --- | --- | --- | --- | --- | --- | --- | --- | --- | --- | --- | --- | --- | --- |
| No. | | *A* | *B* | *C* | *α* | | *β* | *E_b_* | | | *d^a^* | | *D^a^* |
| 1 | | 1.563 | 12052 | 0 | 0.100 | | 0.529 | 7.540 | | | 2.0 | | 168.0 |
| 2 | | 1.554 | 9200 | 0 | 0.100 | | 0.404 | 6.478 | | | 2.0 | | 157.3 |
| 3 | | 1.537 | 9452 | 0 | 0.100 | | 0.269 | 7.107 | | | 2.0 | | 152.4 |
| 4 | | 1.568 | 12650 | 0 | 0.100 | | 0.534 | 9.165 | | | 2.0 | | 161.7 |
| 5 | | 1.567 | 12378 | 0 | 0.100 | | 0.603 | 7.608 | | | 2.0 | | 160.3 |
| 6 | | 1.556 | 11142 | 0 | 0.100 | | 0.315 | 8.893 | | | 2.0 | | 158.4 |
| 7 | | 1.541 | 9440 | 0 | 0.100 | | 0.212 | 10.039 | | | 2.0 | | 180.3 |
| 8 | | 1.542 | 9616 | 0 | 0.100 | | 0.266 | 8.284 | | | 2.0 | | 175.8 |
| Avg. | | 1.554 | 10741 | 0.000 | 0.10 | | 0.392 | 8.139 | | | |  |  |
| Std. dev. | | 0.012 | 1473 |  |  | | 0.148 | | 1.178 | | |  |  |

*^a^d, D* is thickness in *nm*.


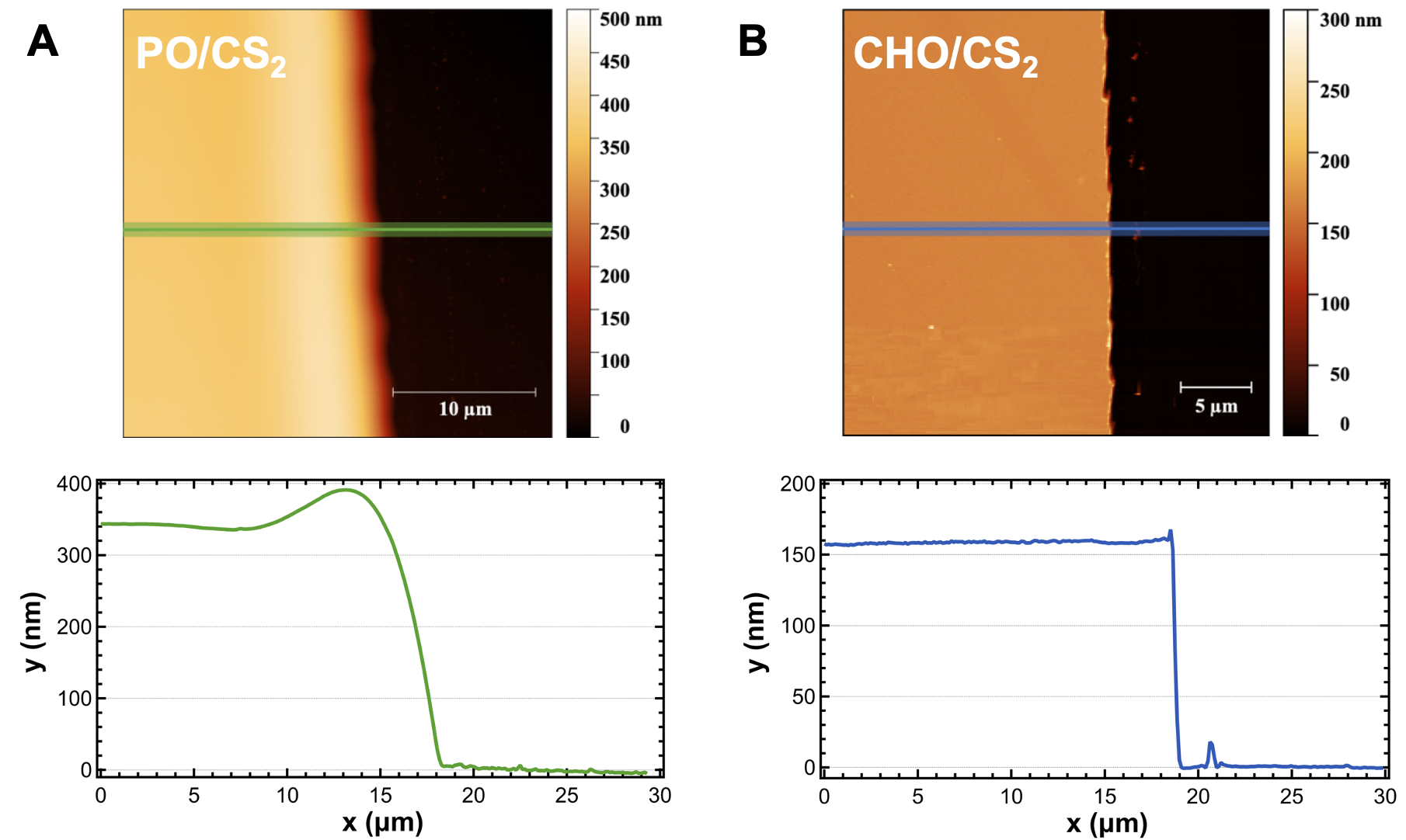


**Supplementary Figure 23.** (**A**) and (**B**) (*Top*) 2D AFM height sensor scans of the scored edge of PO/CS_2_ and CHO/CS_2_ coatings, respectively. (*Bottom*) Line profile plots obtained by averaging ten-line scans, highlighted by the shaded regions in the 2D height sensor scans.
